# Behavior of the OptiVol2 fluid index and intrathoracic impedance on remote monitoring As a detector of subclinical device infection early after implantation

**DOI:** 10.1002/joa3.13005

**Published:** 2024-03-23

**Authors:** Daisuke Togashi, Kenichi Sasaki, Tomoo Harada, Yoshihiro J. Akashi

**Affiliations:** ^1^ Division of Cardiology, Department of Internal Medicine St. Marianna University School of Medicine Kawasaki Japan

**Keywords:** device infection, implantable cardioverter defibrillator, intrathoracic impedance, OptiVol2 fluid index, remote monitoring

## Abstract

We report the behavior of OptiVol2 fluid index (OVFI2) and intrathoracic impedance on remote monitoring before the appearance of signs of infection. A sustained rise in OVFI2 early after implantation reflects peri‐device fluid retention.

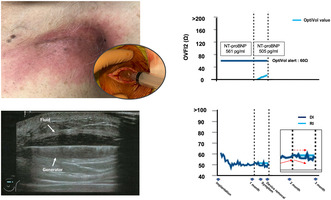

The OptiVol (Medtronic, Minneapolis, MN, USA) fluid index 2.0 (OVFI2) derived from the intrathoracic impedance (ITI) has been used as a parameter to predict heart failure (HF), but false‐positive cases have been reported.^1^ The ITI uses the tip of the right ventricular lead and the generator as the measurement circuit and reflects the state of the fluid volume in the thorax. The DI fluctuates because of factors like posture or breathing during measurement, hence it is measured 64 times a day to calculate its average value. For deriving the OVFI from the ITI, the differences between the RI and DI below the RI are totally summed. The ratio of the ITI at an alert to baseline ITI <0.96 or OVFI >60 Ω is often used as a marker of worsening HF. These two parameters have also been applied as remotely monitored values for predicting HF.^2^ However, there have been several reports of false‐positive alerts with these indexes because of fluctuations caused by an abnormal fluid density in the thoracic region, such as a pocket hematoma or breast reconstruction.^3^, ^4^ The OVFI2, which is the modified algorithm of the OVFI, has several changes compared to the OVFI, such as adjusting the RI under the right circumstances and more rigorously assessing the accumulation of fluid index by subtracting a correction value from the difference between the RI and DI.^1^, ^5^ Nevertheless, the OVFI2 could provide wrong alerts. In this study, we report two cases of subclinical device infection indicated by elevated OVFI2 on remote monitoring.

## 1 CASE 1

**FIGURE 1:**
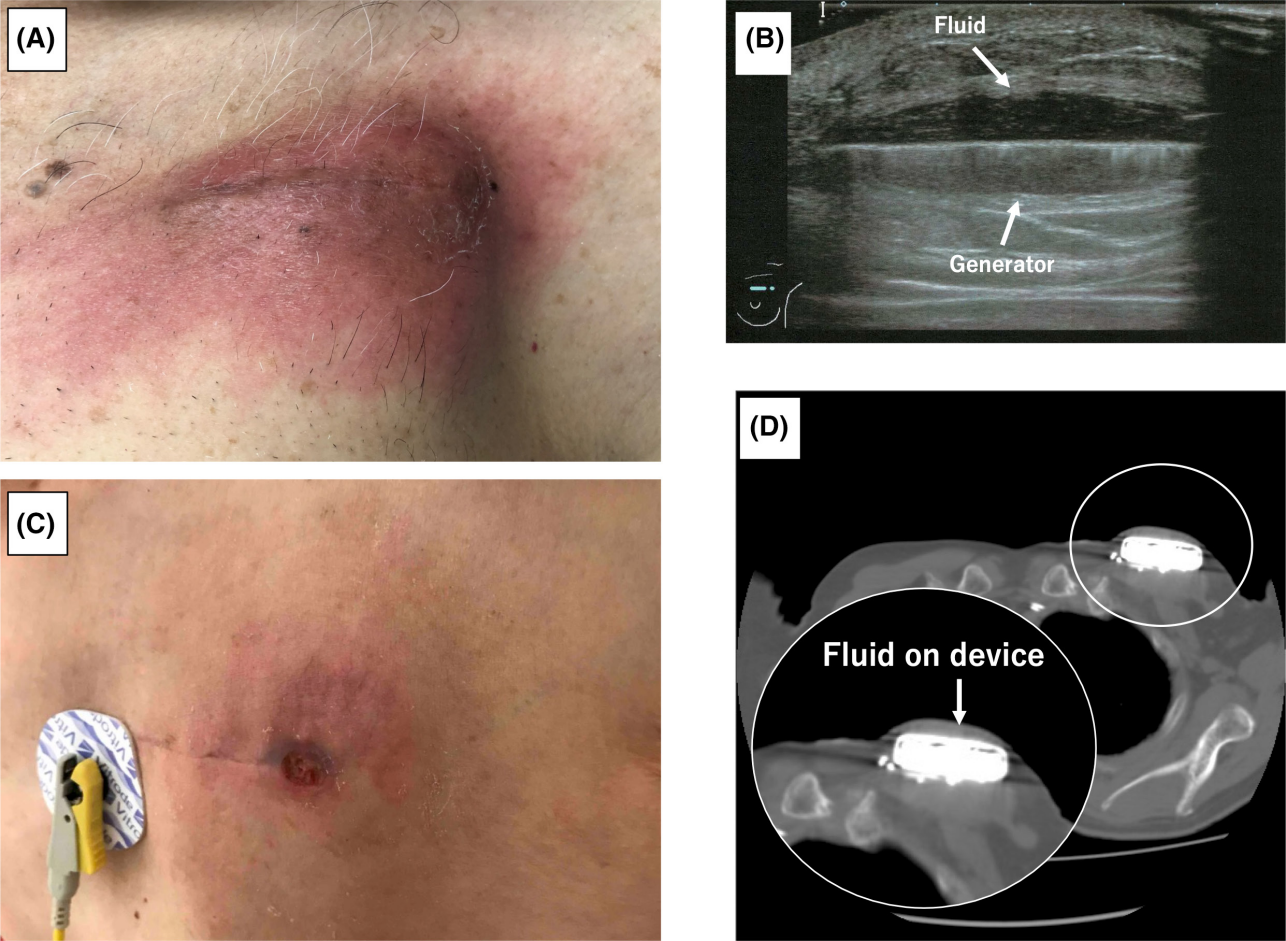
Images of the skin and sonographic or computed tomographic findings around the device‐implanted sites in Case 1 (A and B) and Case 2 (C and D).

**FIGURE 2:**
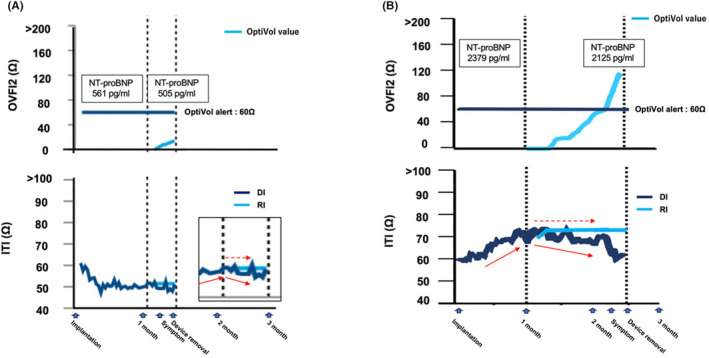
Trends in the OVFI2 and ITI from the remote monitoring in Cases 1 (A) and 2 (B) with a device infection shortly after the implantation. The details are shown in the text. DI, daily intrathoracic impedance; ITI, intrathoracic impedance; OVFI2, OptiVol fluid index 2.0; RI, reference intrathoracic impedance.

A 50‐year‐old man treated with immunosuppressive medications for rheumatoid arthritis and cardiac sarcoidosis underwent catheter ablation because of a sustained ventricular tachycardia (VT). Subsequently, a transvenous implantable cardioverter defibrillator (TV‐ICD) was implanted for secondary prevention of sudden cardiac death. At the 1‐month follow‐up visit, no signs of HF or a device infection were observed. One week after the outpatient clinic, however, he experienced erythema and swelling at the site of the ICD implantation (Figure 1A). Forty‐two days after the implantation, the patient was admitted to our hospital because his symptoms did not improve. Surface sonography showed fluid accumulation around the device (Figure 1B), and blood and wound cultures detected methicillin‐resistant *Staphylococcus aureus*. The ICD system was completely removed by simple traction the day after admission, and a new TV‐ICD was reimplanted on the opposite side after the infection had settled. The remote monitoring data during this period showed that the daily ITI (DI) initially decreased, but soon increased gradually and decreased again after 1 month (blue line in the lower panel of Figure 2A), while the reference ITI (RI) exhibited a plateau (light blue line in the lower panel of Figure 2A). As a result, the OVFI2 continued to increase from the beginning of the measurement (light blue line in the upper panel of Figure 2A). However, the N‐terminal pro‐brain natriuretic peptide (NT‐proBNP) level on admission of 505 pg/mL was the same level as that at pre‐implantation (561 pg/mL); therefore, worsening of HF was unlikely.

## 2 CASE 2

A 78‐year‐old man with dilated cardiomyopathy and a non‐sustained VT underwent a TV‐ICD implantation for primary prevention. Around 70 days after the implantation, the patient presented with a fever and erythema on the ICD pocket (Figure 1C). Laboratory data revealed a markedly elevated inflammatory response and computed tomography showed fluid accumulation on the device (Figure 1D). Blood cultures tested positive for methicillin‐sensitive *S. aureus*. The device system was completely removed the next day. The remote monitoring data showed that the DI increased until the first month after the implantation, then gradually decreased (blue line in the lower panel of Figure 2B), while the RI presented a flat line (light blue line in the lower panel of Figure 2B). As a result, the OVFI2 increased rapidly since the measurement started and reached the threshold (60 Ω) just before the onset of the symptoms (light blue line in the upper panel of Figure 2B). However, the NT‐proBNP value on admission was 2125 pg/mL, which was comparable to the value before the implantation (2379 pg/mL).

## 3 DISCUSSION

**FIGURE 3:**
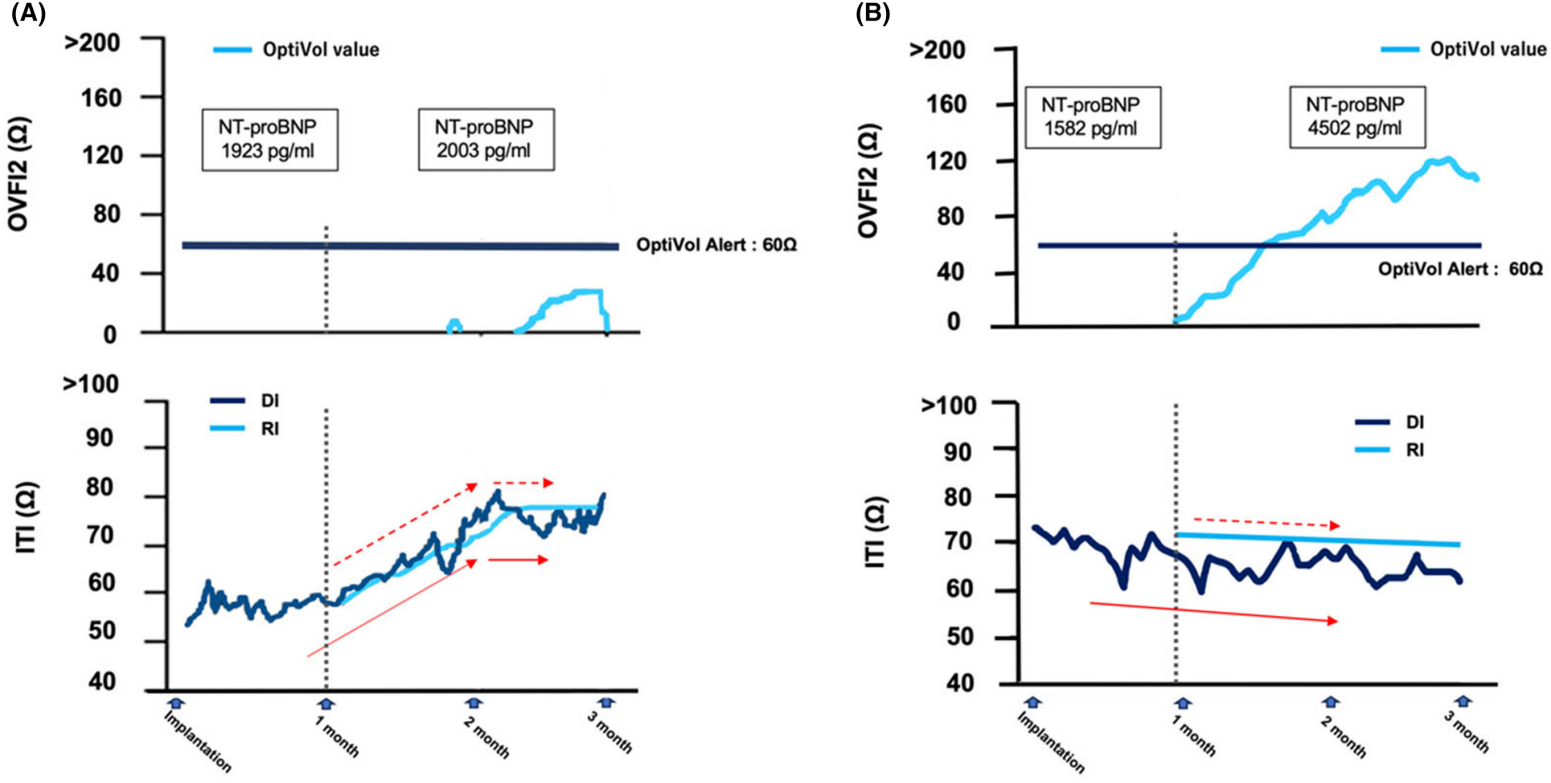
Trends in the OVFI2 and ITI from the remote monitoring in a case with neither a device infection nor HF (A) and a case with HF (B). The details are shown in the text. HF, heart failure. Other abbreviations are as in Figure 2.

Generally, the DI gradually increases and stabilizes 1 month after implantation, as postsurgical hematoma or edema around a device gets absorbed. In our two cases, however, the DI began to decrease after 1 month and was dissociated from the RI trends. These DI behaviors led to an upward trend in the OVFI2 values, which could lead to false‐positive HF events. We experienced two other representative cases: a patient with neither a device infection nor HF (Figure 3A) and a patient with HF (Figure 3B). In the former case, the value of the DI started to increase after the implantation and continued to rise until 2 months later, while the value of the RI also increased concurrently with the DI. Therefore, the OVFI2 remained low. In the latter case, the DI exhibited a downward trend with a day‐to‐day variability from the very beginning as compared to the RI, which had a smooth downward slope. Therefore, the OVFI2 gradually accumulated and rose above the threshold. This pattern could be observed when preexisting HF worsens after implantation. If HF event newly occurs after implantation, it could be difficult to distinguish from device infection by our diagnostic method. This is the limitation of our study. The time courses in these two cases seemed to differ from those in the two above‐mentioned cases with early device infections. In summary, a DI with upward‐to‐downward sloping beyond 1 month after the implantation and a sustained increasing OVFI2 on remote monitoring might be one of the indicators of a subclinical early device infection, particularly in patients with a high risk of device infection, such as malnutrition, immunosuppressant use or history of diabetes. Not only the OVFI2 alerts but also the DI and RI trends should be considered for differentiating HF‐related events from other events.

## FUNDING INFORMATION

None of the authors has any financial disclosures.

## CONFLICT OF INTEREST STATEMENT

The authors declare no conflict of interest for this article.

## ETHICS STATEMENT

Not applicable.

## PATIENT CONSENT STATEMENT

The patient has provided consent for publication.

## CLINICAL TRIAL REGISTRATION

Not applicable.

## References

[1] Miyoshi A , Nishii N , Kubo M , Okamoto Y , Fujii S , Watanabe A , et al. An improved algorithm calculated from intrathoracic impedance can precisely diagnose preclinical heart failure events: sub‐analysis of a multicenter MOMOTARO (monitoring and management of OptiVol alert to reduce heart failure hospitalization) trial study. J Cardiol. 2017;70:425–431. 28673507 10.1016/j.jjcc.2017.05.004

[2] Yu CM , Wang L , Chau E , Chan RH , Kong SL , Tang MO , et al. Intrathoracic impedance monitoring in patients with heart failure: correlation with fluid status and feasibility of early warning preceding hospitalization. Circulation. 2005;112:841–848. 16061743 10.1161/CIRCULATIONAHA.104.492207

[3] Barth C , Kindermann I , Mahfoud F , Buob A , Böhm M . Intrathoracic impedance monitoring detecting pneumonia. Clin Res Cardiol. 2010;99:333–335. 20165857 10.1007/s00392-010-0127-9

[4] Nagra B , Ibrahim T , Shah AN , Kantharia BK . Persistently abnormal device‐detected thoracic impedance and OptiVol fluid index related to breast reconstruction surgery. Pacing Clin Electrophysiol. 2023;46:323–326. 36272170 10.1111/pace.14610

[5] Sarkar S , Hettrick DA , Koehler J , Rogers T , Grinberg Y , Yu CM , et al. Improved algorithm to detect fluid accumulation via intrathoracic impedance monitoring in heart failure patients with implantable devices. J Card Fail. 2011;17:569–576. 21703529 10.1016/j.cardfail.2011.03.002

